# Derivation of a new parametric impulse response matrix utilized for nodal wind load identification by response measurement

**DOI:** 10.1016/j.jsv.2014.12.027

**Published:** 2015-05-26

**Authors:** A. Kazemi Amiri, C. Bucher

**Affiliations:** a Vienna Doctoral Programme on Water Resource Systems, Karlsplatz 13/222, A-1040 Vienna , Austria; b Center for Mechanics and Structural Dynamics, Karlsplatz 13/206, A-1040 Vienna, Austria

## Abstract

This paper provides new formulations to derive the impulse response matrix, which is then used in the problem of load identification with application to wind induced vibration. The applied loads are inversely identified based on the measured structural responses by solving the associated discrete ill-posed problem. To this end — based on an existing parametric structural model — the impulse response functions of acceleration, velocity and displacement have been computed. Time discretization of convolution integral has been implemented according to an existing and a newly proposed procedure, which differ in the numerical integration methods. The former was evaluated based on a constant rectangular approximation of the sampled data and impulse response function in a number of steps corresponding to the sampling rate, while the latter interpolates the sampled data in an arbitrary number of sub-steps and then integrates over the sub-steps and steps. The identification procedure was implemented for a simulation example as well as an experimental laboratory case. The ill-conditioning of the impulse response matrix made it necessary to use Tikhonov regularization to recover the applied force from noise polluted measured response. The optimal regularization parameter has been obtained by L-curve and GCV method. The results of simulation represent good agreement between identified and measured force. In the experiments the identification results based on the measured displacement as well as acceleration are provided. Further it is shown that the accuracy of experimentally identified load depends on the sensitivity of measurement instruments over the different frequency ranges.

## 1 Introduction

The load identification in engineering problems becomes more important if the excitations are caused by the actions which cannot be measured directly. A good knowledge on applied loads is necessary for extraction of their characteristics or their reproduction via simulation for other purposes. The idea of direct measurement of the applied loads generally becomes more infeasible if the structure as well as the load action become more complex. For example, wind loading is one of the dominant design parameters for structures having low natural frequencies such as tall buildings or long bridges. The presence of the fluctuating wind components due to turbulence makes the wind force measurement even tougher, because more sensitive force or pressure measurement devices in huge numbers are necessary, which might not be practically realistic.

On the other hand measurement of the response to the excitation is more common and better developed due to its extensive use in the other application areas such as system identification. Consequently an indirect procedure that gives the ability to identify the load from measured response — a so-called inverse problem — seems to be attractive. Load identification can be done in time or frequency domain, the best choice depends on the type of loading or the identification purposes. The authors׳ interest is to apply the solution of this inverse problem, i.e. the inverse wind load identification, in wind fatigue analysis of a full-scale guyed-mast. Therefore it is required to prepare and evaluate a load identification procedure in the time domain, which is consistent with the methodology in the subsequent application.

For the sake of load identification we initially need to set up a complete input–output relation for the direct problem (based on already known, e.g. previously identified parameters of the structure) for two reasons. Firstly having an updated finite element model for fatigue analysis is needed. Secondly utilization of an experimental input–output model such as quasi-impulse response matrix [1] due to its limitations is not appropriate.

Once the input–output model is generated the load identification can formally be treated in the discrete manner as a linear system of equations that is, $u=\overset{¯}{H}p$. Unfortunately, it is an ill-posed problem since the impulse response matrix $\overset{¯}{H}$ is usually ill-conditioned and the deconvolution by means of pseudo-inverse multiplication may lead to unbounded solutions in the presence of noise in measured data u. Consequently we need to call upon alternative methods which are designed to solve discrete ill-posed problems. From a general aspect these methods are classified into direct or iterative procedures. However, neither regularization by means of projection of the problem nor the iterative regularization plus projection (c.f. [2]) falls within the scope of this paper. Thus the solution of a discrete ill-posed problem just based on a direct scheme is dealt with. In this scheme, there are several different ways such as Tikhonov regularization [3] and family of truncated singular value decomposition (TSVD) methods [4,5]. These methods aim at filtering out the contribution of noise in the response or improving the conditioning of $\overset{¯}{H}$.

It must be noted that the regularization methods need a regularization parameter as a stop criterion to tune the amplitude of smoothening of the response. The parameter selection methods are categorized into two general classes. The first class includes the methods working based on a priori knowledge about the measurement noise and the second class, which is applicable independently. The methods of generalized cross validation (GCV) [6] and L-curve [7,8] are two examples of the second class. Also it has been recommended to evaluate which parameter selection method is more efficient according to the nature of the certain problem.

In this paper the regularization method of Tikhonov for the regularization together with two procedures of GCV and L-curve for finding the regularization parameter were selected. We provide the results of load identification via simulation and laboratory experiments for a rigidly clamped cantilever beam. The applied loads are realized by white noise limited to 25 Hz and wind excitation, which are identified separately from measured displacement and acceleration response by means of the derived ordinary and augmented impulse response matrices.

## 2 Dynamic response analysis of discrete-time systems

In this section an input–output relation in a discretized time domain is constructed. In other words we are going to build the impulse response matrix, which when multiplied by the input signal (i.e. force record) renders the output as time history of displacement. In this paper this aim was reached by means of modal analysis since it is closely consistent with modal testing methods of system identification in practical cases. It is mentioned here that the input–output relation might also be alternatively derived via testing, mathematical system identification [9] or simulation of application of impulsive loads on degrees of freedom when the finite element model of the structure exists.

### 2.1 Impulse response matrix

The linear equations of motion for an MDOF system with classical damping are given in the following system of differential equations:

(1) $$m\overset{¨}{u}+c\overset{˙}{u}+\mathrm{ku}=p(t)$$

while u, m, c, k denote, the displacement, mass, damping and stiffness of the system respectively, as well as the dynamic force p, which applies on the system׳s degrees of freedom.

Projecting Eq. (1) onto modal coordinates by means of substitution u(t)=Φq(t) together with premultiplying each term in the equation by $\Phi^{T}$ renders the set of uncoupled modal equations of motion [10]:

(2) $$\overset{¨}{q}+2\mathrm{diag}[\zeta_{i}\omega_{i}]\overset{˙}{q}+\mathrm{diag}[\omega_{i}]q=P(t)$$

Each single equation in the system of Eq. (2) may be solved by means of a convolution integral (Duhamel׳s integral) and the response in all modal coordinates in a compact form is

(3) $$[\begin{array}{c} q_{1}\\ \vdots \\ q_{n} \end{array}]=\int_{0}^{t}[\begin{array}{ccc} h_{1}(\tau ) & & 0\\ & \ddots & \\ 0 & & h_{n}(\tau ) \end{array}]\{\begin{array}{c} P_{1}(t-\tau )\\ \vdots \\ P_{n}(t-\tau ) \end{array}\}d\tau =\int_{0}^{t}h(\tau )\Phi^{T}p(t-\tau )\;d\tau$$

where *n* denotes the number of degrees of freedom. The impulse response function h(τ) can be calculated mathematically by solving the SDOF equation of motion. Then we move backward and incorporate the responses to all vibration modes i.e. computing the superimposed response in the global coordinates. Hence Eq. (3) is premultiplied by Φ which yields the response in the global coordinates:

(4) $$u=\int_{0}^{t}\underset{\overset{¯}{h}}{{\underset{︸}{\Phi h(\tau )\Phi }}^{T}}p(t-\tau )\;d\tau$$

For numerical evaluation of the convolution integral, $\overset{¯}{h}(\tau )$ and p(t−τ) might be assumed to be constant within the time step. This approximation becomes very inaccurate especially when the loading record includes high frequency components. Then the discrete-time-domain response becomes [11]

(5) $$u\approx \mathrm{dt}\overset{k-1}{\underset{j=0}{\sum }}{\overset{¯}{h}}_{j}p_{k-j-1}$$

while *k* and dt stand for the total number of time steps and the length of that, respectively. At each time step the matrix of impulse response functions is computed as $\overset{¯}{h_{j}}=\overset{¯}{h}(j\;\mathrm{dt})$ and $p_{k-j-1}$ refers to the discretized force at the time step k−j−1. The final task in creating the displacement impulse response matrix namely $\overset{¯}{H}$ is rearranging the previous equation in the matrix form. The resulting impulse response matrix due to its ordinary integral scheme is called ${\overset{¯}{H}}_{\mathrm{Ord}}$:

(6) $$\left[\begin{array}{c} {\left\{\begin{array}{c} u_{1}\\ \vdots \\ u_{n} \end{array}\right\}}_{1}\\ \vdots \\ {\left\{\begin{array}{c} u_{1}\\ \vdots \\ u_{n} \end{array}\right\}}_{k} \end{array}]=\underset{{\overset{¯}{H}}_{\mathrm{Ord}}}{\underset{︸}{\mathrm{dt}[\begin{array}{cccc} {\overset{¯}{h}}_{0} & & & 0\\ {\overset{¯}{h}}_{1} & {\overset{¯}{h}}_{0} & & \\ \vdots & \vdots & \ddots & \\ {\overset{¯}{h}}_{k-1} & {\overset{¯}{h}}_{k-2} & \cdots & {\overset{¯}{h}}_{0} \end{array}]}}[\begin{array}{c} {\left\{\begin{array}{c} p_{1}\\ \vdots \\ p_{n} \end{array}\right\}}_{0}\\ \vdots \\ {\left\{\begin{array}{c} p_{1}\\ \vdots \\ p_{n} \end{array}\right\}}_{k-1} \end{array}\right]$$

#### 2.1.1 Augmented impulse response matrix

**Fig. 1:**
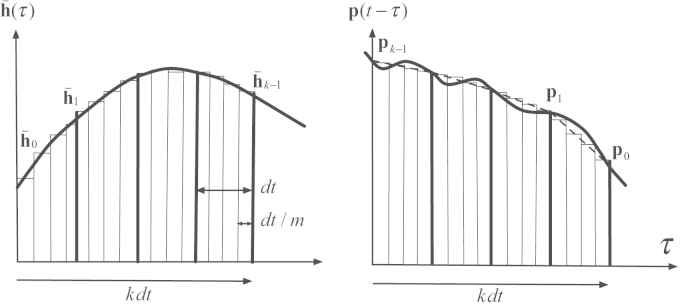
Evaluation of convolution integral considering the force interpolation.

For a better accuracy of numerical methods for approximating the dynamic response a small time step is necessary. Unfortunately, selection of a smaller time step leads to a larger $\overset{¯}{H}$ and accordingly larger size of problem in load identification. In parallel with growth of size of problem the regularization becomes computationally more demanding. Therefore another way of improving the efficiency of $\overset{¯}{H}$ was sought in which the size of problem is kept constant. In this regard we introduce *augmented* impulse response matrix, which is generated by means of linearly interpolating the forces (sampled discrete values) between consecutive steps in an arbitrary number of sub-steps. The discretization pertaining to the augmented impulse response matrix is depicted in Fig. 1.

After applying the trapezoidal rule, the discretized displacement response is computed by means of augmented impulse response in the following form:

(7) $$\left[\begin{array}{c} {\left\{\begin{array}{c} u_{1}\\ \vdots \\ u_{n} \end{array}\right\}}_{0}\\ \vdots \\ {\left\{\begin{array}{c} u_{1}\\ \vdots \\ u_{n} \end{array}\right\}}_{k} \end{array}]=\underset{{\overset{¯}{H}}_{\mathrm{Aug}}}{\underset{︸}{\frac{\mathrm{dt}}{2m^{2}}[\begin{array}{ccccc} 0 & & \cdots & & 0\\ {\overset{¯}{H}}_{1,1} & {\overset{¯}{H}}_{1,2} & & & \\ {\overset{¯}{H}}_{2,1} & {\overset{¯}{H}}_{2,2} & {\overset{¯}{H}}_{1,2} & & \vdots \\ \vdots & \vdots & \ddots & \ddots & \\ {\overset{¯}{H}}_{k-1,1} & {\overset{¯}{H}}_{k-1,2} & \cdots & {\overset{¯}{H}}_{2,2} & {\overset{¯}{H}}_{1,2} \end{array}]}}[\begin{array}{c} {\left\{\begin{array}{c} p_{1}\\ \vdots \\ p_{n} \end{array}\right\}}_{0}\\ \vdots \\ {\left\{\begin{array}{c} p_{1}\\ \vdots \\ p_{n} \end{array}\right\}}_{k} \end{array}\right]$$

The augmented impulse response matrix, ${\overset{¯}{H}}_{\mathrm{Aug}}$, consists of the components ${\overset{¯}{H}}_{u,v}=\Phi \;\mathrm{diag}[{h_{u,v}}_{1}\cdots {h_{u,v}}_{n}]\Phi^{T}$ while $h_{u,v}$ denotes the element of the following matrix at *n*-th mode:

(8) $$h_{n}=[\begin{array}{ccccc} a_{1,1} & b_{1,2} & & \cdots & 0\\ a_{2,1} & c_{2,2} & b_{2,3} & & \\ \vdots & \vdots & \ddots & \ddots & \vdots \\ a_{k-1,1} & c_{k-1,2} & \cdots & c_{k-1,k-1} & b_{k-1,k} \end{array}]$$

The components of $h_{n}$ are obtained as

(9a) $$a_{j,1}=\overset{m-1}{\underset{p=0}{\sum }}(2p+1)h_{j+(2p-1)/2m-1}$$

(9b) $$b_{j,j+1}=\overset{m-1}{\underset{p=0}{\sum }}(2(m-p)-1)h_{(2p-1)/2m}$$

(9c) $$c_{j,k+1}=\overset{j-1}{\underset{k=0}{\sum }}(\overset{m-1}{\underset{p=0}{\sum }}(2p+1)h_{j+(2p-1)/2m-2}+\overset{2m-1}{\underset{p=m}{\sum }}(4m-2p-1)h_{j-k+(2p-1)/2m-2})$$

### 2.2 Complete set of discrete dynamic response

For a full formulation of input-out setup, the impulse response functions of displacement, velocity and acceleration should be known. Afterwards depending on the type of the response sensor attached to any degrees of freedom, the applied force may be inversely identified.

The impulse response function of the displacement is a well-known function and available in a couple of publications. Thus the impulse response functions of velocity and acceleration for a single mode need to be determined. These functions were calculated by solving the equation of motion of an SDOF system under impulsive force and are given through Eqs. (10) for t≥0:

(10a) $$h=\frac{e^{-\zeta \omega_{n}t}}{m\omega_{d}}\sin\omega_{d}t$$

(10b) $$\overset{˙}{h}=\frac{e^{-\zeta \omega_{n}t}}{m\omega_{d}}[\omega_{d}\cos\omega_{d}t-\zeta \omega_{n}\sin\omega_{d}t]$$

(10c) $$\overset{¨}{h}=\frac{1}{m}[\delta (t)-\frac{e^{-\zeta \omega_{n}t}}{\omega_{d}}(2\zeta \omega_{n}\omega_{d}\cos\omega_{d}t+\omega_{n}^{2}\sin\omega_{d}t)]$$

where δ(t) denotes the Dirac delta function.

With the similar reasoning to reach Eq. (4) and using the corresponding function from Eqs. (10), the dynamic response of velocity and acceleration can be calculated. Consequently the complete set of responses of an *n* degrees of freedom system for an excitation interval discretized in *k* time steps is available by using any discretization scheme, as below:

(11a) $${\left\{u\right\}}_{k⁎n}={\left[{\overset{¯}{H}}_{d}\right]}_{\left(k⁎n)(k⁎n\right)}{\left\{p\right\}}_{k⁎n}$$

(11b) $${\left\{\overset{˙}{u}\right\}}_{k⁎n}={\left[{\overset{¯}{H}}_{v}\right]}_{\left(k⁎n)(k⁎n\right)}{\left\{p\right\}}_{k⁎n}$$

(11c) $${\left\{\overset{¨}{u}\right\}}_{k⁎n}={\left[{\overset{¯}{H}}_{a}\right]}_{\left(k⁎n)(k⁎n\right)}{\left\{p\right\}}_{k⁎n}$$

These relations might be reduced in the absence of either any response sensors or excitations in a single node.

## 3 Load identification using regularization method

There is a couple of methods dealing with providing a good solution for the system of linear equations $u_{\mathrm{poll}}=\overset{¯}{H}p$ when the matrix $\overset{¯}{H}$ is ill-conditioned and the vector $u_{\mathrm{poll}}$ is polluted by noise. Here we use *Tikhonov* regularization method [3], which has the following form:

(12) $$\min\{{∥u_{\mathrm{poll}}-\overset{¯}{H}p∥}^{2}+\alpha^{2}{∥p∥}^{2}\}$$

The norm sign here as well as in the subsequent equations denotes the *Euclidean norm*. The difficulty with solving the optimization problem Eq. (12) is how to choose the appropriate parameter α (referred to as regularization parameter) because the solution is sensitive to its choice.

### 3.1 Selection of regularization parameter

#### 3.1.1 L-curve

L-curve is the log–log plot of the smoothened solution versus the residual norm, corresponding to different values of regularization parameter. Depending on the selection of regularization parameter, there is a trade-off between residual norm and the size of solution. Hence L-curve aims at finding the balancing regularization parameter, which should lie on the corner of L-curve. This corner can be approximately recognized as the point on which the curvature of L-curve is maximal. As a result, the problem of parameter selection changes to finding such an *α*, which is the minimizer of negative value of L-curve׳s curvature [8].

#### 3.1.2 GCV

This method suggests us to solve the following constrained optimization problem for load identification to restrict the norm of load response:

(13) $$\min\{\frac{1}{n}{∥u_{\mathrm{poll}}-\overset{¯}{H}p∥}^{2}+\lambda {∥\left(p\right)∥}^{2}\}$$

while *n* is the number of rows of $\overset{¯}{H}$. It could be interpreted as Tikhonov regularization in which α is chosen as $\sqrt{n\lambda }$.

This method approximates the regularization parameter as the minimizer of the function [6]

(14) V(λ)=1n∥(I−H¯H¯^(λ))upoll∥2/[1nTrace(I−H¯H¯^(λ))]2

while H¯^(λ)=(H¯TH¯+nλI)−1H¯T is the matrix, which maps $u_{\mathrm{poll}}$ onto the solution p. In other words p is the solution of minimization expression in Eq. (13) i.e. p=H¯^upoll

## 4 Numerical results

In the first step the solutions of direct problem, i.e. dynamic response analysis, by means of ordinary and augmented response matrices are compared. These results, provided in Section 4.1, aim at justifying the introduction of augmented impulse response matrix.

Secondly we provide the numerical results of inverse problem in terms of simulation and experimental laboratory-scale load identification, respectively in Sections 4.2 and 4.3. The inverse problem has been solved by means of regularization methods.

The case study is a rigidly clamped aluminum alloy cantilever, which has been modelled as a single degree of freedom system. As a result there is always one unknown force versus a single measured structural response. The system parameters of the equivalent mass–spring–dashpot model corresponding to the cantilever have been identified, which are given in Section 4.3.1. The identified system parameters were utilized to construct the parametric impulse response matrices for numerical and experimental load identification in Sections 4.2 and 4.3, respectively.

The codes needed for the procedure of load identification have been implemented in slangTNG [12]. The results gained from slangTNG were cross-checked with those achieved from Regularization toolbox [13], which showed good agreement with each other.

The accuracy of the identified load, $p_{\mathrm{ident}.}$, with respect to the actual force i.e. $p_{\mathrm{act}.}$ is evaluated by the following definition:

(15) $$\mathrm{Error}(\%)=∥p_{\mathrm{ident}.}-p_{\mathrm{act}.}∥/∥p_{\mathrm{act}.}∥⁎100$$

### 4.1 Response comparison of ordinary and augmented schemes

**Fig. 2:**
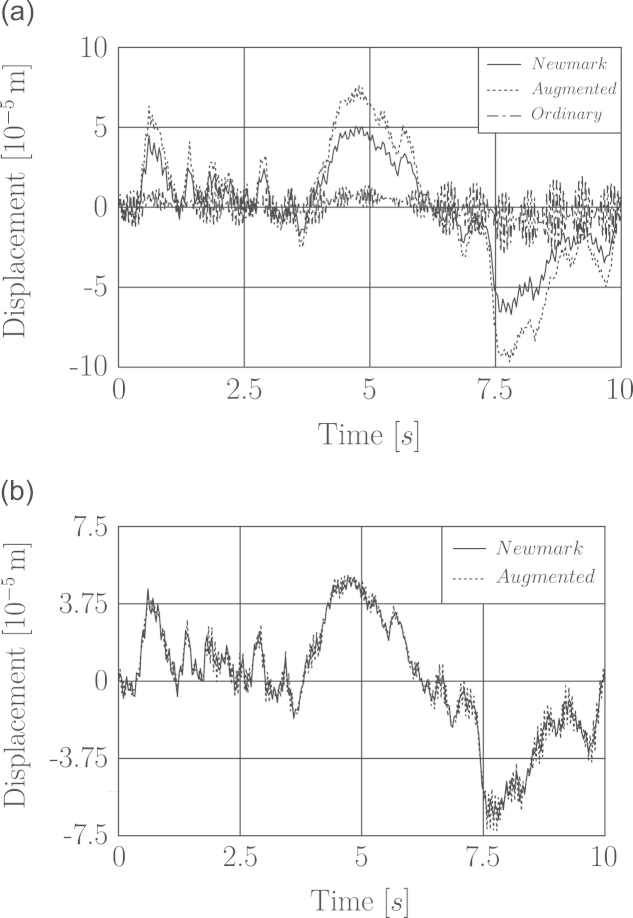
Effect of increasing number of sub-steps in augmented scheme response of structure to wind load excitation. Number of augmented scheme sub-steps (a) *m*=1 and (b) *m*=5.

It was already stated in Section 2.1.1 that the computed structural response by augmented scheme has more accuracy over the ordinary scheme. Our investigations show that by increasing the number of sub-steps in augmented scheme its corresponding response is improved. This leads to the faster convergence of augmented scheme compared to the ordinary one in computing the dynamic response, thus consequently provides a smaller sized problem in the load identification. In Fig. 2 we demonstrate the displacement response under wind loading computed by ordinary and augmented impulse response matrix in comparison with the Newmark method response as the benchmark method. In this example the structure is a single degree of freedom system with the same specifications as those of the laboratory case study given in Section 4.3.1. In wind load simulation the data time step was taken to be 0.1 s. The time step to calculate the displacement response was set to 0.034 s. Therefore the wind force data was linearly interpolated to obtain the same time step length. The number of sub-steps in computing the displacement response based on augmented scheme is 1 and 5. The comparison between the computed response of augmented scheme in Fig. 2(a) and (b) indicates that in a given time step length with increasing number of sub-steps an accurate response by augmented scheme is obtained. On the other hand the response from ordinary impulse matrix given in Fig. 2(a) has not still converged, which means that the length of time step for ordinary scheme has yet to be shortened. It has been observed that the response by augmented scheme changes very slightly for sub-steps m>5.

### 4.2 Simulation of load identification

In order to simulate the measured structural response, white noise was added to the computed “displacement responses” of the structure under the actual force. Then this noise-polluted displacement response was used for the load identification purpose. The magnitude of the additional noise was scaled with respect to the standard deviation of the actual response and adjusted by defining a noise level multiplier. In the numerical simulation of load identification, the number of sub-steps for construction of augmented impulse response matrix i.e. *m* is considered to be 5.

**Fig. 3:**
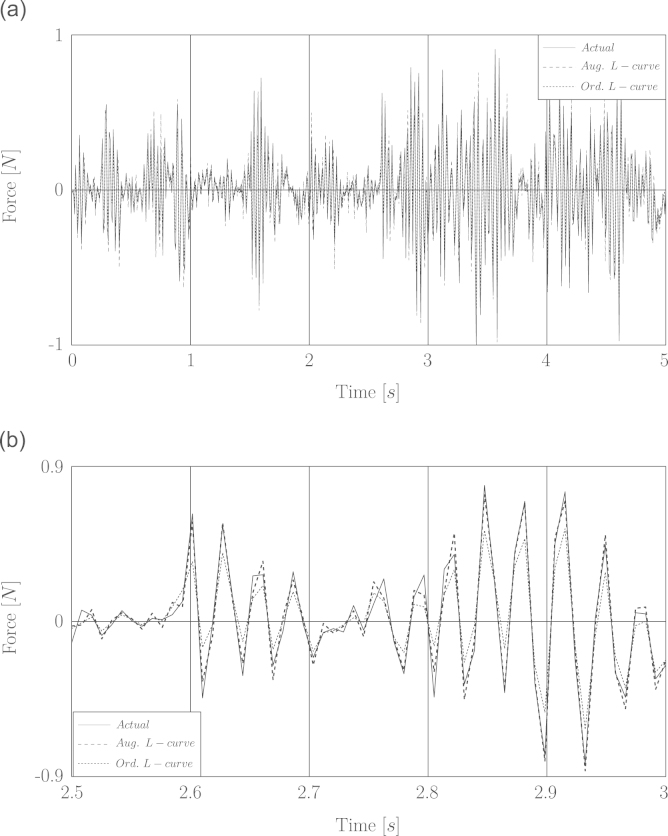
Simulation of load identification based on noisy displacement for White noise excitation. (a) Full time history. (b) A window of time history between 2.5 and 3 s.

Firstly the recovering of a white noise excitation limited to 25 Hz is simulated. The duration of excitation, noise level and sampling rate are 5 s, 2.5 percent and 120 s^−1^ respectively. The simulation of identification was run several times and it was observed that both identification methods are stable in recovering the force based on the noisy displacement response. Use of the word “stable” means that the optimal regularization parameter can be found based on the criteria of L-curve or GCV as many as times the load identification was repeated. Moreover the identified forces obtained by using the augmented impulse response matrix were recovered more accurately than those obtained by using the ordinary impulse response matrix (c.f. Table 1). Fig. 3 represents the comparison of the identified white noise with the actual force.

In order to evaluate the consistency of identification methods for wind load, the fluctuating parts of wind velocity and correspondingly wind loads were generated. This was done by simulating a one dimensional single variable stationary random process, thus just the wind velocity autospectrum is needed. For digital simulation of wind speed, amongst existing power spectral density (PSD) functions [14], Davenport׳s autospectrum [15] was (arbitrarily) selected. The spectrum was widened and the upper frequency was extended to 30 Hz in order to cover the relatively high resonance frequency region of the structure. The wind force PSD is plotted in Fig. 8.

For simulation of wind load identification, the actual displacement response of the structure was polluted at 2.5 percent noise level. The response sampling rate and duration of excitations were respectively 180 s^−1^ and 5 s.

The error analysis of the identified wind loads is given in Table 1. The results demonstrate that the recovered wind force by use of augmented impulse response matrix via L-curve is unacceptable. This problem occurs due to the difficulty in finding the location of the L-curve corner, since many points on the L-curve have the properties to be the optimal regularization parameter based on L-curve criterion.

The recovered wind forces via GCV using both impulse response matrices have the same quality and are better identified than their counterpart by means of L-curve and ordinary impulse response matrix.

**Fig. 4:**
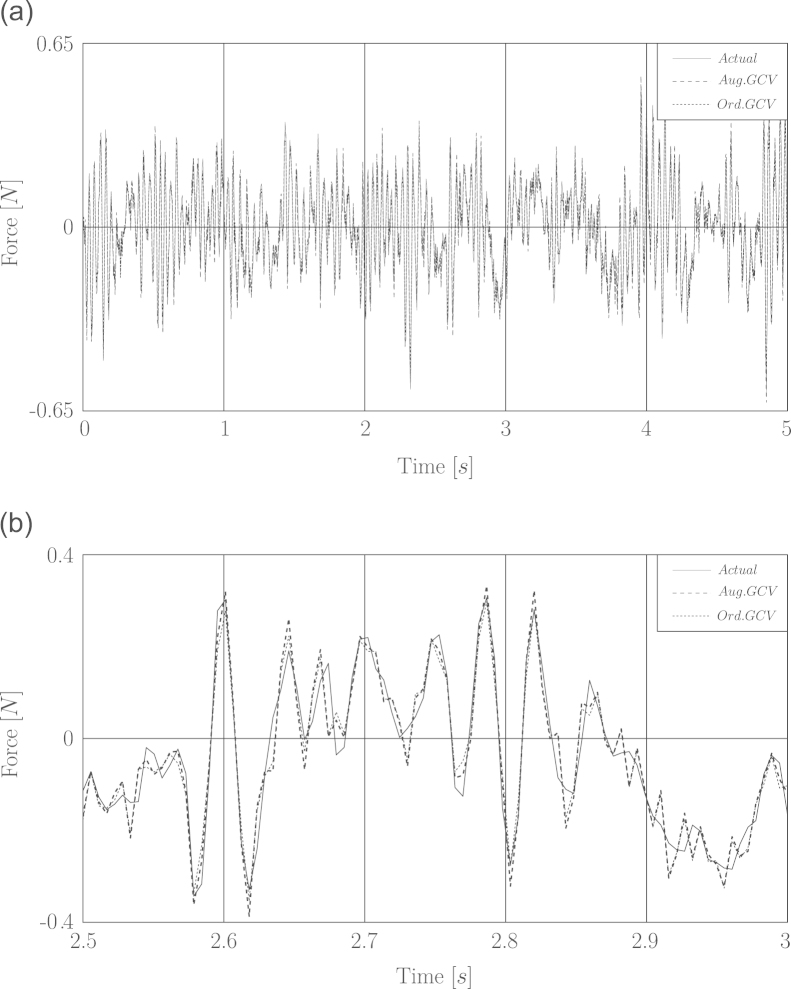
Simulation of load identification based on noisy displacement for fluctuating wind load. (a) Full time history. (b) A window of time history between 2.5 and 3 s.

In Fig. 4 the time history of recovered wind forces based on the noisy displacement response is shown.

### 4.3 Laboratory-scale load identification

#### 4.3.1 Case study

**Fig. 5:**
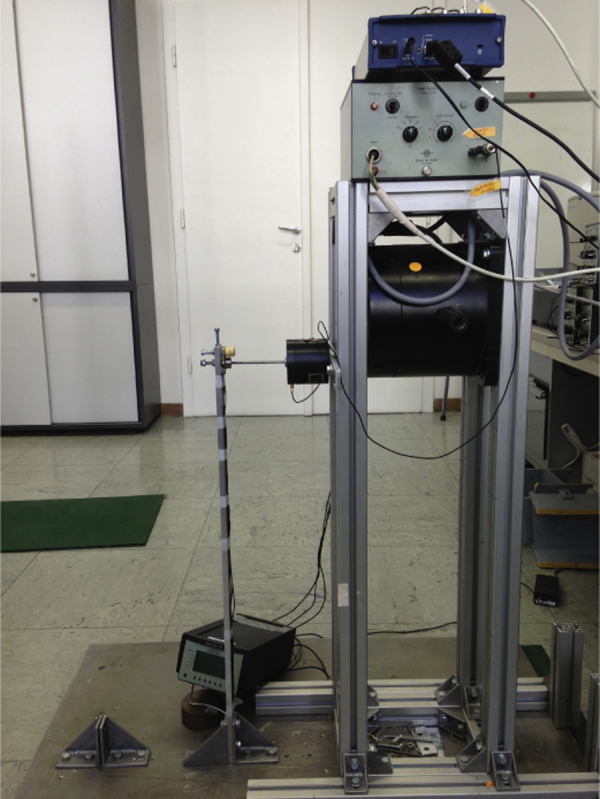
Picture of laboratory-scale setup.

As mentioned earlier the case study investigates the load identification for a vertically erected aluminum alloy cantilever whose one end is rigidly clamped. The cantilever is a rectangular beam with cross sectional dimensions 0.03 and 0.01 m as well as 0.68 m height, which is bending over its weak axis. Three sensors have been mounted on the tip of the beam. Two of them are piezoelectric accelerometers, which are used to derive the acceleration and displacement responses at the same time. The displacement response was obtained from the measured acceleration via the signal conditioner. The third sensor measures the applied force directly from the shaker. Shaker applies one point-force at the tip of the beam as well. The specifications of measurement equipments, which are the products of Brüel & Kjær, are given in Table 2. The picture of the whole setup is also shown in Fig. 5.

The configuration of loading stimulates the structure such that the first mode is the governing mode of vibration. As a result the structure has been modeled directly as a single degree of freedom system and the dynamic response of the cantilever is truncated to its first mode. Then we readily determined the first mode eigenfrequency, mass, stiffness and damping ratio. Firstly the eigenfrequency was determined by an impulse test and compared with those gained via incremental-frequency and variable-frequency harmonic excitation that was estimated nearly the same as 13.8 Hz. By successively attaching lumped masses on the cantilever׳s tip and observing the change in the eigenfrequencies, the equivalent mass and stiffness were determined, while the damping ratio was obtained via harmonic decay test. The modal system parameters including mass, stiffness and damping ratio are respectively 0.2196 kg, 1666.4 N m^−1^ and 0.07 percent.

#### 4.3.2 Experimental load identification

For the sake of experimental load identification the identification procedure was repeated for the white noise and fluctuating wind force based on measured displacement. Moreover in this section the results of the experimental wind load identification based on measured acceleration will be also provided. The acceleration responses have been used just when we initially speculated that the reason of inaccuracy in the identified wind load is related to the measured displacement response. However the main focus is on using the measured displacement response, since the L-curve or GCV function corresponding to the measured displacements was seen to be better shaped than those of the acceleration response. The investigation on application of the acceleration-based wind load identification will be dealt with in more detail in the forthcoming lines.

The first part of the experimental analysis again consists of identification of the white noise excitation with the same features as previously explained in Section 4.2. The results of experimentally recovered white noise show nearly the same accuracy with which were obtained in the corresponding numerical simulation. The identification results of white noise are provided in Table 3. Since the identification of white noise excitation has the satisfactory exactness alike the simulations, we end with it here and resume with the wind load identification.

**Fig. 6:**
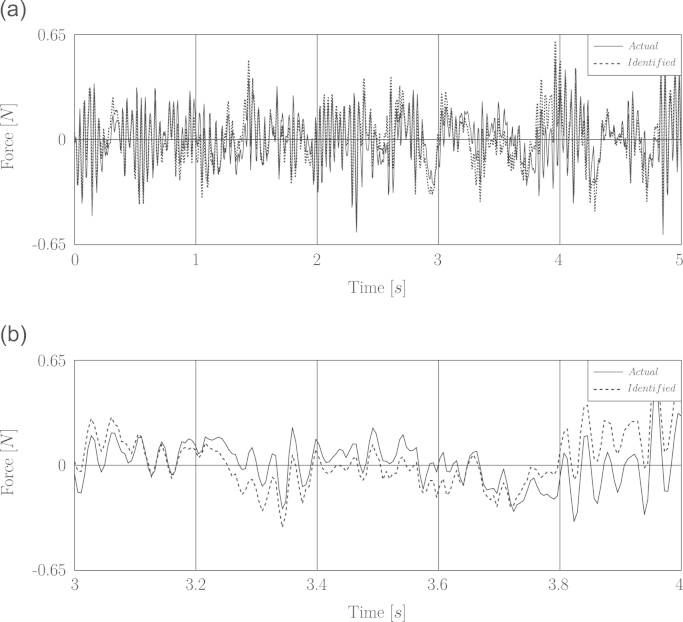
Experimentally identified fluctuating wind load based on measured displacement response. (a) Full time history. (b) A window of time history between 3 and 4 s.

The experimental wind load identification was repeated based on the measured displacement response as have been already simulated in Section 4.2 for wind-type excitation. The identification results of wind load demonstrate that the recovered forces based on GCV or L-curve criteria do not have acceptable accuracy. For instance in Fig. 6 the comparison of identified fluctuating wind load with the actual one by means of GCV in augmented scheme has been depicted. Table 3 provides the wind load identification results based on different methods in both schemes. According to this table the errors corresponding to the augmented scheme are slightly smaller than the ordinary one but nevertheless not good enough.

With repeating the procedure of the experimental wind force recovering under different simulated wind excitations, it was perceived that the regularization methods are stable while the recovered force is not in agreement with the measured force. It means that the optimal regularization parameters found based on the GCV and L-curve criteria do not render the identified loads of good quality.

First of all it was supposed that this problem occurs due to the high-pass filtering within double integration of acceleration response, since we have obtained the displacement from acceleration response via the signal conditioner device. Thus in order to circumvent such problem, the wind load was identified directly based on the acceleration response by selecting the third of Eqs. (10). Several runs of this procedure show that slightly better or worse results may be achieved. Therefore the utilization of acceleration response does not significantly improve the identified wind load.

**Fig. 7:**
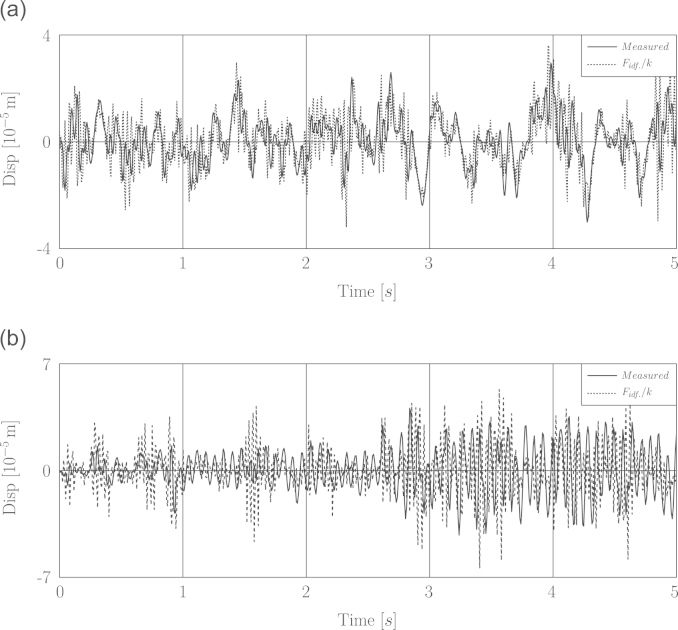
Comparison between measured and quasi-static displacements. (a) Fluctuating wind excitation. (b) White noise excitation.

More inspection revealed that the displacement-based identified load divided by structure׳s stiffness, *k*, is very close to the measured displacement response. This comparison was illustrated in Fig. 7(a). But rather as shown in Fig. 7(b), this analogy does not exist for the white noise excitation case. Hence according to this observation, it can be concluded that the use of this kind of measured displacement response led to recovering a sort of quasi-static force. In other words the measured displacements due to the wind excitation, which has dominant low frequency components, do not sufficiently contain the portion of high frequency components of the displacement response. As a result, those parts of loading concerning the inertia and damping force are not identified.

According to this finding it was assumed that the inadequate sensitivity of the measurement sensors and not other issues regarding the identification methods gave rise to the inaccurate identified wind loads. It was because the applied wind excitation has the characteristics that stimulated the stiffness of the structure (which belongs to lower frequency components of response) much more than its inertia (which conversely belongs to higher frequency components of response). In addition, the portion of the damping force due to the small damping ratio of structure as well as the low velocity of loading is not considerable.

One way to resolve the problem in measurement when the sensors do not have sufficient sensitivity is to amplify the amplitude of the quantity of interest, which in our case is the acceleration response. This amplification must occur before the sensor level so that the sensor be able to sense it. Therefore the excitation input must be modified. Since the acceleration response is proportional to the excitation frequency, consequently by raising the lower band of excitation frequency we can increase the amplitude of acceleration response.

**Fig. 8:**
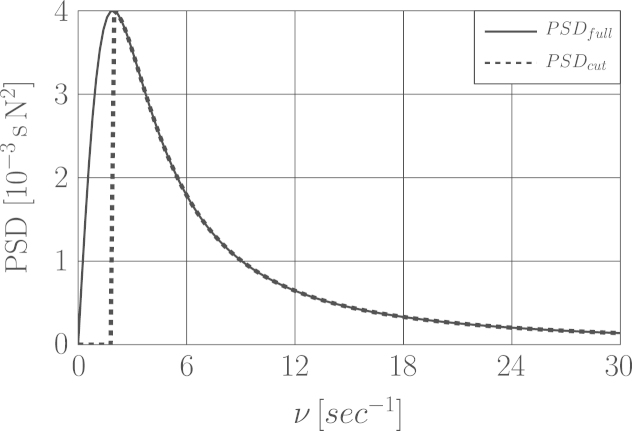
Wind force autospectral densities for full frequency range and one with $\nu_{\mathrm{cut}\;\mathrm{off}}=[02.5]$ Hz.

To this end we filtered out different frequency ranges in the lower band of the wind power spectral density by definition of three cut-off frequency ranges i.e. $\nu_{\mathrm{cut}\;\mathrm{off}}$ for [02.5], [05] and [07.5] Hz. For example the corresponding wind spectrum to $\nu_{\mathrm{cut}\;\mathrm{off}}=[0\;2.5]$ is depicted in Fig. 8.

**Fig. 9:**
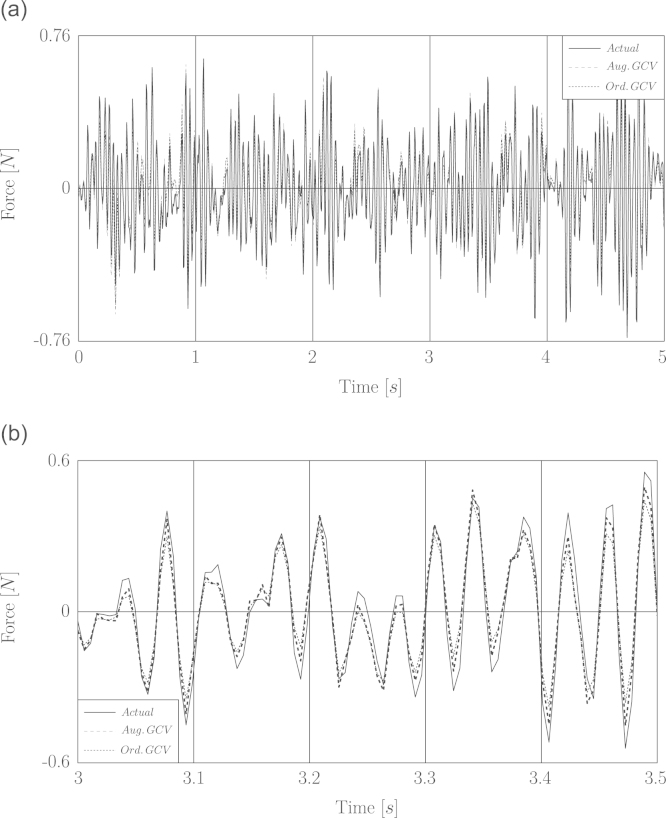
Experimentally identified fluctuating wind load with $\nu_{\mathrm{cut}\;\mathrm{off}}$ between [02.5] Hz based on measured displacement response. (a) Full time history. (b) A window of time history between 3 and 3.5 s.

The identification results associated with the newly simulated wind forces not only prove our second assumption but also shows that the major problem was resolved when the very low frequency excitation components i.e. between [02.5] Hz were filtered out. Because the accuracy of the identified newly generated wind loads was considerably improved by elimination of this range. It was observed that for the other two cut-off ranges the improvement is insignificant. The recovered wind load with $\nu_{\mathrm{cut}\;\mathrm{off}}=[0\;2.5]$ Hz by means of GCV is illustrated in Fig. 9. The results of identified wind loads based on measured displacement for wind loads with full frequency range as well as those with $\nu_{\mathrm{cut}\;\mathrm{off}}$ are provided in Table 4.

The problem of identification in low level vibrations could be also resolved either by use of acceleration transducer, which are appropriate for such vibrations or by means of strain gauge instead of accelerometer as reported in [16].

**Fig. 10:**
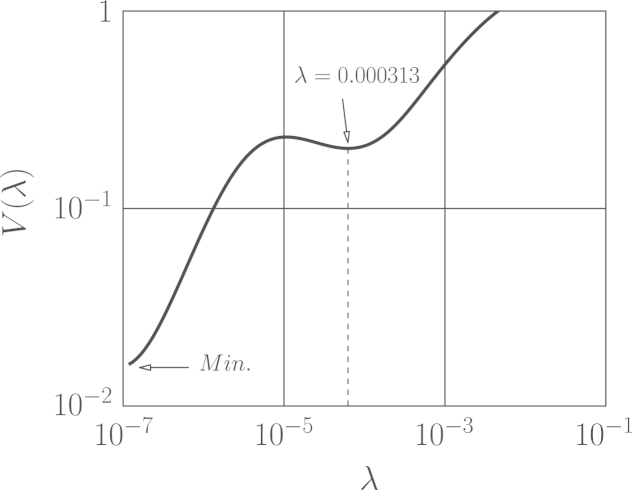
GCV minimizer in identifications by means of measured acceleration.

The same sets of newly generated wind excitations has been also used for force recovery by means of measured accelerations. Several times within wind load identification by means of measured accelerations, some irregularities in the shape of the curvature of L-curve or GCV minimizer have been observed that violated the L-curve and/or GCV criteria in finding the optimal regularization parameter. For instance, in Fig. 10 the GCV minimizer of the wind load with $\nu_{\mathrm{cut}\;\mathrm{off}}=[0\;2.5]$ Hz corresponding to the ordinary impulse matrix is demonstrated. This figure reveals despite GCV׳s criterion, the proper identified solution is associated with the GCV parameter, which is located at the local minimum rather than the global minimum. The associated error of identified wind loads based on measured acceleration responses are presented in Table 4. In this table the superscript-marked values correspond to the cases that the optimal regularization parameters located at the local minimum. The trouble in these cases justifies to use the displacement response in load identification since the L-curve or GCV function corresponding to the measured displacements was nearly always well-shaped.

## 5 Conclusions

In this paper the formulations for derivation of impulse response matrices, which are used in the de-convolution problem of load identification, were presented. The dynamic loads were identified based on structural response measurement and by means of solving the corresponding inverse problem.

Construction of two different types of impulse response matrices (ordinary and augmented), which are different in their integration schemes, was also presented. According to Section 4.1 the response via the augmented scheme converges faster than the response of the ordinary one. This is because the ordinary impulse response matrix is constructed based on the constant approximation of force within a time step. This assumption demands such a small time step in order that the approximated force resembles the real one.

Consequently in order to be able to select the time step longer than that of the ordinary scheme the augmented impulse response matrix, which interpolates the force in a number of sub-steps was introduced. However in the context of accuracy comparison, the sampling rates were selected relatively high so that the response of the ordinary scheme can converge too. Nonetheless the forces recovered by means of the augmented impulse matrix were more accurate than by the ordinary one even for such small time step lengths (c.f. Tables 1 and 4).

The identification results illustrate that the high accuracy of load identification drastically depends on the use of appropriate response sensors for different types of excitation in the sense of the contained frequency range. Otherwise the recovered force accuracy would be poor such as it occurred for the recovered wind load as was for instance shown in Fig. 6.

Last but not least the load identification based on the measured displacement response was observed to be more robust than acceleration based when L-curve or GCV methods are used. This is due to the fact that the L-curve and GCV criteria corresponding to the acceleration response were observed to fail way more often than when the displacement response was utilized. As a result there is the lack of robustness in the placement of the optimal regularization parameter according to those criteria (e.g. Fig. 10) when the acceleration response is used.

## Figures and Tables

**Table 1:** Error (%) associated with the identified loads via simulation.

Impulse response matrix	White		Wind force	
	L-curve	GCV	L-curve	GCV
Ordinary	34	35	38	29
Augmented	23	21	–⁎	29

⁎ Has not resulted to an acceptable response.

**Table 2:** Laboratory equipments specifications.

Type	Sensitivity	Frequency range (kHz)	Measurement utility
Accelerometer 4383	3.217 (pC/ms^−2^)	0.1–8.4	Displacement
Accelerometer 4383	3.190 (pC/ms^−2^)	0.1–8.4	Acceleration
Force transducer 8230	3.915 (pC/N)	–⁎	Force
Nexus 2692	–	0.1–100	Signal conditioner

⁎ Has not been provided by the manufacturer.

**Table 3:** Error (%) associated with the experimental identified loads.

Impulse response matrix	White		Wind force	
	L-curve	GCV	L-curve	GCV
Ordinary	31	32	61	61
Augmented	21	21	60	57

**Table 4:** Error (%) associated with the experimental identified loads based on measured displacement and acceleration

Cut-off range (Hz)	Impulse matrix	Measurement			
		Displacement		Acceleration	
		L-curve	GCV	L-curve	GCV
–	Aug.	60	57	36⁎	44
	Ord.	61	61	50	48
[0 2.5]	Aug.	34	31	31	34
	Ord.	36	37	35	36⁎
[0 5]	Aug.	33	29	31	29
	Ord.	35	36	35	31⁎
[0 7.5]	Aug.	31	27	31⁎	31
	Ord.	34	34	34	33⁎

⁎ Corresponding to the regularization parameter at local minima.

## References

[1] Ł. Jankowski, Dynamic load identification for structural health monitoring, IPPT Report on Fundamental Technological Research 2/2013, Institute of Fundamental Technological Research, Polish Academy of Sciences, Warsaw, 2013.

[2] Klimer M., O’Lary D.P. (2001). Choosing regularization parameter in iterative methods for ill-posed problems. SIAM Journal on Matrix Analysis and Applications.

[3] Tikhonov A.N., Arsenin V.Y. (1997). Solution of Ill-posed Problems.

[4] Varah J.M. (1973). On the numerical solution of ill-conditioned linear systems with application to ill-posed problems. SIAM Journal on Numerical Analysis.

[5] Hansen P.C. (1987). Regularization, gsvd and truncated gsvd. BIT.

[6] Wahba G., Golub G.H., Heath M. (1979). Generalized cross-validation as a method for choosing good ridge parameter. Technometrics.

[7] Lawson C.L., Hanson R.J. (1974). Solving Least Squares Problems.

[8] Hansen P.C., O’Lary D.P. (1993). The use of L-curve in the regularization of discrete ill-posed problems. SIAM Journal on Scientific Computing.

[9] Juang J. (1994). Applied System Identification.

[10] Ziegler F. (1998). Mechanics of Solids and Fluids.

[11] L. Meirovitch, *Computational Methods in Structural Dynamics*, Sijthoff and Noordhoff International Publishers, Alphen aan den Rijn, 1980.

[12] C. Bucher, S. Wolff, *SlangTng: A Toolkit for Stochastic Mechanics Analysis*, Vienna University of Technology (2007–2013).

[13] Hansen P.C. (2007). A matlab package for analysis of discrete ill-posed problems. Numerical algorithms.

[14] Simiu E., Scanlan R.H. (1978). Wind Effects on Structures.

[15] Davenport A.G. (1961). The spectrum of horizontal gustiness near the ground in high wind. Quarterly Journal of the Royal Meteorological Society.

[16] B. Hillary, D.J. Ewins, The use of strain guages in force determination and frequency response function measurements, *Proceedings of the 2nd International Modal Analysis Conference IMAC*, FL, USA, 1984, pp. 627–634.

